# Sex-Specific Behavioral Response to Early Adolescent Stress in the Genetically More Stress-Reactive Wistar Kyoto More Immobile, and Its Nearly Isogenic Wistar Kyoto Less Immobile Control Strain

**DOI:** 10.3389/fnbeh.2021.779036

**Published:** 2021-12-14

**Authors:** Sarah Kim, Stephanie A. Gacek, Madaline M. Mocchi, Eva E. Redei

**Affiliations:** Department of Psychiatry and Behavioral Sciences, Feinberg School of Medicine, Northwestern University, Chicago, IL, United States

**Keywords:** Wistar Kyoto More Immobile, passive coping, depression, social recognition, sex differences, brain-derived neurotrophic factor, glucocorticoid receptor

## Abstract

Genetic predisposition and environmental stress are known etiologies of stress-related psychiatric disorders. Environmental stress during adolescence is assumed to be particularly detrimental for adult affective behaviors. To investigate how genetic stress-reactivity differences modify the effects of stress during adolescence on adult affective behaviors we employed two inbred strains with differing stress reactivity. The Wistar Kyoto More Immobile (WMI) rat strain show increased stress-reactivity and despair-like behaviors as well as passive coping compared to the nearly isogenic control strain, the Wistar Kyoto Less Immobile (WLI). Males and females of these strains were exposed to contextual fear conditioning (CFC) during early adolescence (EA), between 32 and 34 postnatal days (PND), and were tested for the consequences of this mild EA stress in adulthood. Early adolescent stress significantly decreased anxiety-like behavior, measured in the open field test (OFT) and increased social interaction and recognition in adult males of both strains compared to controls. In contrast, no significant effects of EA stress were observed in adult females in these behaviors. Both males and females of the genetically less stress-reactive WLI strain showed significantly increased immobility in the forced swim test (FST) after EA stress compared to controls. In contrast, immobility was significantly attenuated by EA stress in adult WMI females compared to controls. Transcriptomic changes of the glucocorticoid receptor (*Nr3c1*, GR) and the brain-derived neurotrophic factor (*Bdnf*) illuminate primarily strain and stress-dependent changes, respectively, in the prefrontal cortex and hippocampus of adults. These results suggest that contrary to expectations, limited adolescent stress is beneficial to males thru decreasing anxiety and enhancing social behaviors, and to the stress more-reactive WMI females by way of decreasing passive coping.

## Introduction

Adolescence has gained increasing attention as a sensitive period of development, a period in which pubertal transitions may increase the vulnerability to stressors (McCormick et al., 2017). Adolescence is defined as a transition between childhood and adulthood, and puberty is thought to be an important hallmark of it. Adolescence is also of great clinical importance; it is the time in which many mental health problems such as mood disorders emerge. Further, the risk of adult drug abuse and addiction is greater in adults who were exposed to drugs in their adolescence, rather than those who started drug use in adulthood (Odgers et al., 2008). Animal studies has confirmed the effect of stress during adolescence to be deleterious for adult affective and cognitive functions as well as stress reactivity (Pohl et al., 2007; McCormick et al., 2008; Arnett et al., 2015). However, these effects of adolescent stress were found to be sex dependent (McCormick and Mathews, 2007; Weintraub et al., 2010). It is often thought that females are more vulnerable to the effects of adolescent stress, particularly in their depressive behavior (Mathews et al., 2008; Bourke and Neigh, 2011). In contrast, differences have been observed in anxiety-like behaviors of adult males after acute adolescent stress, with no or very limited effect in females (Lovelock and Deak, 2019).

In animal studies, stressors can be administered to rodents at postnatal day (PD) 22–32 during early adolescence (EA) or peripubertal period, later in adolescence up to PD 45, or post-pubertally (Lo Iacono and Carola, 2018). Stress experienced in the peripubertal phase is of clinical importance, as it is associated with the development of disorders such as depression, anxiety, and post-traumatic stress disorder in adulthood (Spear, 2000; Bale et al., 2010; McCrory et al., 2012; Turecki, 2012; Brown and Spencer, 2013). It is known that acute stress produces more prolonged activation of the hypothalamic-pituitary-adrenal (HPA) axis in peripubertal animals than in adults, thus, the consequence of acute stress during this developmental period could be long lasting (Lui et al., 2012). However, how genetic predisposition to increased stress-reactivity interacts with a mild developmental challenge is less explored. Specifically, animals of differing stress-reactivity that encounter a mild, acute stress during the prepubertal EA period may produce lesser or differential affective behaviors in adulthood.

Two inbred rat strains with differential stress-reactivity were employed in this study. These strains were generated by bidirectional selection from the Wistar Kyoto (WKY) parental strain using immobility behavior in the forced swim test (FST) as a functional selector (Will et al., 2003). The by now inbred Wistar Kyoto More Immobile (WMI) strain consistently show greater immobility compared to the inbred WKY Less Immobile (WLI) strain (Andrus et al., 2012; Mehta et al., 2013). Adult WMI males have decreased anxiety-like behavior in the open field test (OFT) compared to WLI males, while females of both strains have shown the same high levels of anxiety in adulthood (Mehta et al., 2013). WMI males also show greater behavioral stress reactivity than WLI males, indicated by their increased fear memory after stress (Lim et al., 2018).

Our hypothesis was that EA stress would exacerbate the behavioral deficits of WMI rats and generate a vulnerability in the WLI strain in adulthood. Additionally, we hypothesized that the genetic differences in stress-reactivity between the strains would interact with the sex differences observed in other rodent studies in adult behaviors after EA stress. To test these hypotheses, males and females of the more stress-reactive WMI, and their nearly isogenic less stress-reactive control WLIs were exposed to a stressor during early adolescence. This stressor was a mild foot-shock received during the contextual fear conditioning (CFC) test. In adulthood, we tested remote fear memory (RM) to determine the EA stress effects. After RM, we tested affective behaviors that are known to differ between the strains and sexes in adult WMI and WLIs. Previously, strain and sex differences have been seen in the OFT and in the FST, the latter being the functional selector for the original selective breeding (Will et al., 2003; Mehta et al., 2013). After adult stress, social interaction and recognition also differed within the strains by sex (Schaack et al., 2021).

Expression of the brain-derived neurotrophic factor (BDNF) has been shown to be region-specifically responsive to stress and thought to be a marker of neuronal plasticity (Bath et al., 2013). The BDNF stress-sensitivity hypothesis posits that disruption in the endogenous BDNF activity potentiates sensitivity to stress (Notaras and van den Buuse, 2020). Since the WMI strain show enhanced sensitivity to stress, *Bdnf* expression in relevant brain regions, specifically the hippocampus and the prefrontal cortex, has been studied. Although the nature of the BDNF-glucocorticoid connection is very complex (Choy et al., 2008; Numakawa et al., 2013), a brain region and development-dependent connection has been suggested between them (Choy et al., 2008; Daskalakis et al., 2015). Therefore, we examined the glucocorticoid receptor (*Nr3c1*) expression in the same brain regions.

The purpose of this study was to determine whether genetic hypersensitivity to stress would alter behaviors in adulthood after a limited mild stress during early adolescence. Additionally, sex differences in the effects of EA stress could also be determined, and thereby ascertain the significance of sex in the consequences of genetic—environmental interactions.

## Materials and Methods

### Animals

Animal procedures were approved by the Institutional Animal Care and Use Committee of Northwestern University. The Wistar Kyoto (WKY) More Immobile (WMI) rat strain, a genetic rat model of enhanced stress-reactivity and depressive-like behavior, and the Wistar Kyoto Less Immobile (WLI) control strain are derived from the WKY parental strain. The inbred WMI and the nearly isogenic inbred WLI animals were of the 39–41st generation. Due to the low fecundity of these animals (Luo et al., 2020), animals from multiple generation were used to achieve the numbers needed for the study. The rats were maintained at Northwestern University Feinberg School of Medicine by the Center for Comparative Medicine. Animals were group housed (2–3 per cage), maintained on a 12-h light and dark cycle (lights on at 0600h) in a temperature and humidity-controlled room with *ad libitum* access to food and water.

Adolescent male and female WMI and WLI rats were used (*n* = 7–8/sex/strain) in the stress group at postnatal day (PND) 32–34. Control WMI and WLI animals (*n* = 6–7/sex/strain) were not disturbed until adulthood. Adult behavioral testing started at PND 70–75.

Behavioral testing was carried out in the following time sequence: Adolescent FC at PND 32–34; Remote Memory in the CFC at PND 70–75; OFT at PND 77–82; Social interaction (SI) and recognition (SR) at 90–125; FST at PND 120–150, and animals were sacrificed at PND 180–210. The rest periods between the tests were selected to minimalize carryover effects from the previous behavioral tests.

All behavioral experiments and sacrificing the animals were conducted between 1,000 and 1,600 h. Between 1,000 and 1,600 h, there is no major change in the circadian rhythm-driven plasma CORT levels (Solberg et al., 2001; Sage et al., 2004). The behavioral experiments were either recorded and analyzed by the computerized behavioral system, or video recorded, and behaviors were analyzed by trained observers during repeated viewing.

### Behavioral Measures

#### Contextual Fear Conditioning During Adolescence

Male and female, WLI and WMI adolescents (PND 32–34) were placed into an automated fear conditioning apparatus of Technical and Scientific Equipment (TSE, Bad Homburg, Germany) for 3 min of habituation, followed by one foot shock (0.8 mA, 1 s duration). The animals spent 1 min after the shock in the chamber. Twenty-four hours later, the rats were placed in the same apparatus for 3 min, without a shock, and their percent freezing, and total distance traveled were recorded automatically.

#### Remote Contextual Fear Memory in Adulthood

Remote fear memory of the animals was measured between PND 70–75. The animals were placed back into the automated fear-conditioning apparatus for 3 min, without a shock, and their percent freezing, and total distance traveled were recorded.

#### Open Field Test

Control and early adolescent stressed (EA-stressed) animals were tested between PND 77–82, a week after the remote memory test as described. Rats were placed in the center of an 82-cm diameter arena for 10 min. The time spent in the center and distance traveled by the animal were measured in the center 50-cm diameter area by TSE Videomot 2 version 5.75 software (TSE, Bad Homburg, Germany).

#### Social Interaction and Recognition

At PND 90–125, animals were singly housed for 48 h and tested for social interaction and recognition. The SI test employed in this study was selected in order to avoid provoking anxiety in the test animal. Anxiety-like behavior is known to differ between WLI and WMI males and females (Mehta et al., 2013). Therefore, in the present SI test, juveniles were chosen as stimulus animals, because their much smaller size does not threaten the test animals and generate anxiety (Engelmann et al., 1995). The wider than usual age range of the animals was due to the low fecundity of these strains (Luo et al., 2020), and therefore, the infrequent availability of the juvenile stimulus animals.

During the sample trial of the SI test (T1), one 25–28 days old juvenile rat was placed in the home cage of the test animal for 4 min. The juvenile was removed, and the test animal was given a 45-min rest period. This rest period was chosen as the maximum time for social recognition of familiar conspecifics in rats (Squires et al., 2006). After the 45-min rest, the original (familiar) juvenile and a novel juvenile were placed in the home cage with the adult for a 4-min test trial (T2). Juveniles were distinguished by different color non-toxic marks on their tails. Both trials were videotaped from the long side of the home cage, and behaviors were analyzed by trained observers using multiple stopwatches and repeated viewing.

The behavior of the test animals was scored for olfactory investigation (direct contact sniffing and following) in both the sample and test trials. Olfactory investigation was recorded separately for each juvenile and was defined as seconds spent sniffing within 1 cm of each juvenile. Social recognition was characterized by subtracting the time the test animal spent time investigating the familiar juvenile in the test trial from investigating the same juvenile in the sample trial (T2-T1). A larger number indicates that the test animal remembers the familiar juvenile.

#### Forced Swim Test

A minimum of 2 weeks after the social interaction, social recognition test, FST was carried out as described by Porsolt et al. (1977). The adult, 4–5 months old animals were placed into a glass cylinder (30 cm diameter, 45 cm deep) of 23°C tap water for 15 min. Twenty four hours later, rats were again placed into the cylinder of water for 5 min. Activity during the second swim test was video-recorded for subsequent scoring using a time-sampling technique previously described (Detke et al., 1995) in which behavior was scored as immobility, climbing, or swimming every 5 s. We have previously shown that immobility in the FST is not related to body weight (Solberg et al., 2001). Increased immobility indicates increased despair-like behavior, or according to another interpretation increased passive coping.

### Brain Dissection and RNA Isolation

Animals were sacrificed at 6–7 months of age via swift decapitation. Whole brains were removed and stored in RNAlater (Invitrogen, Carlsbad, CA) at room temperature for 24 h and then at –80°C until dissection. Brains were thawed on ice and dissected. Prefrontal cortex and hippocampi were dissected on a brain matrix and immediately stored in RNAlater (Invitrogen, Carlsbad, CA) at –80°C. Paxinos coordinates were used: Prefrontal cortex (AP 5.20–1.70, ML 0–3.3, DV 28 9.0–4.4) and hippocampus (AP –2.12 to –6.0, ML 0–5.0, DV 5.4–7.6) (Wilcoxon et al., 2005).

The tissue was homogenized using TRI Reagent (Sigma-Aldrich, Saint Louis, MO) and a handheld tissue homogenizer (Kinetica Polytronic). Total RNA was isolated from brain samples using the Direct-zol RNA MiniPrep Plus kit (Zymo Research, Irvine, CA) according to manufacturer’s instructions. RNA quality was determined using a NanoDrop (Thermo Fisher Scientific). Accepted quality ranged from 1.8 to 2.2 for 260/280 and 260/230 ratios. Samples were stored at –80°C.

### Reverse Transcription and Quantitative Polymerase Chain Reaction

Reverse transcription was carried out using the Super Script VILO Master Mix (Invitrogen). 1.0 μg of total RNA was used with this manufacturer’s protocol. qPCR was performed with 5 ng cDNA, specific primer pairs for *Nr3c1* (forward: 5′AACAGACTTTCGGCTTCTGGAA 3′; reverse 5′ TGGAACGCTGGTCGACCTAT 3′), and for *Bdnf* (forward: 5′ ATTACCTGGATGCCGCAAAC 3′; reverse 5′ GGGACTTTCTCCAGGACTGT 3′) SYBR Green Master Mix (Applied Biosystems, Foster City, CA, United States), using the QuantStudio™ 6 Flex Real-Time PCR System (Applied Biosystems). Triplicate reactions were performed for each cDNA sample. Relative quantification levels of gene expression, or RQ values, were determined relative to *Gapdh* (forward: 5′ CAACTCCCTCAAGATTGTCAGCAA 3′; reverse: 5′ GGCATGGACTGTGGTCATGA 3′) and a general cDNA calibrator (from blood or hippocampus), using the 2^–ΔΔCt^ method, performed by the QuantStudio™ Software (Applied Biosystems).

### Statistics

All data were represented as mean ± standard error of the mean. All measures were analyzed using a 3-way analysis of variance (ANOVA, stress x strain x sex) followed by false discovery rate (FDR) for multiple comparisons (GraphPad 9.0, San Diego, CA; Benjamini and Hochberg, 1995). Significance was established at *q* ≤ 0.05, but occasionally when the comparison did not reach significance by correction for multiple comparison, individual *p* ≤ 0.05 values were also indicated on the figures. ANOVA results are reported in the results, while *post hoc* significance is shown on the figures. Cohen’s d effect sizes were also calculated, and the ranges are described in the figure legends where it could be compared with the q and p statistics.

## Results

### Behavioral Consequences of Stress During Early Adolescence

**FIGURE 1:**
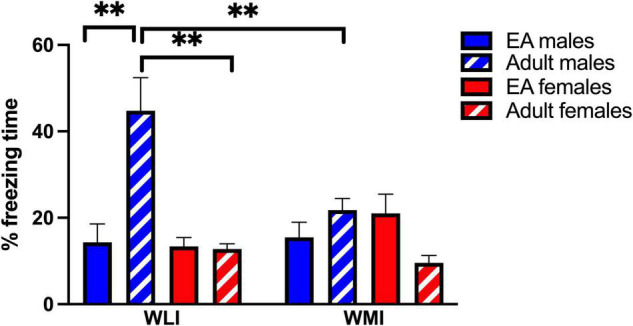
Contextual fear memory of early adolescents and remote memory of adult WLI and WMI males and females expressed as percent freezing. Early adolescent WLI and WMI males and females were tested in the CFC on postnatal day (PND) 32–34. The same animals were placed back into the CFC apparatus as adults and their percent freezing determined without foot-shock for measuring their remote memory. There was no difference in fear memory in early adolescents, but when the animals were returned to the context, WLI males froze significantly more than adult WLI females and WMI males. Data are presented as mean ± standard error of mean (SEM). *N* = 5–8/sex/strain. *Post hoc* comparisons are indicated as ^**^q < 0.01 and determined by two stage linear step-up procedure of Benjamini, Krieger and Yekutieli (FDR) after three-way ANOVA. Cohen’s d effect sizes were 1.77–2.60 in *post hoc* comparisons.

During EA, there were no significant differences in fear memory due to strain or sex as measured by percent freezing (Figure 1). When adults were placed into the CFC without foot-shock to indicate remote memory (RM) after over a month, percent freezing was dramatically and significantly higher in adult WLI males after the EA stress of CFC compared to adolescent WLI males, adult WLI females and adult WMI males [sex × age, *F*(1, 52) = 18.26, *p* < 0.01; strain × age, *F*(1, 52) = 9.35, *p* < 0.01; strain × sex, *F*(1, 52) = 5.28, *p* < 0.05]. This elevated percent freezing of EA-stressed adult WLI males contributed to the significant main effect of sex and age [sex, *F*(1, 52) = 11.99, *p* < 0.01; age, *F*(1, 52) = 4.66, *p* < 0.05]. There was no effect of EA stress on adult females’ RM.

**FIGURE 2:**
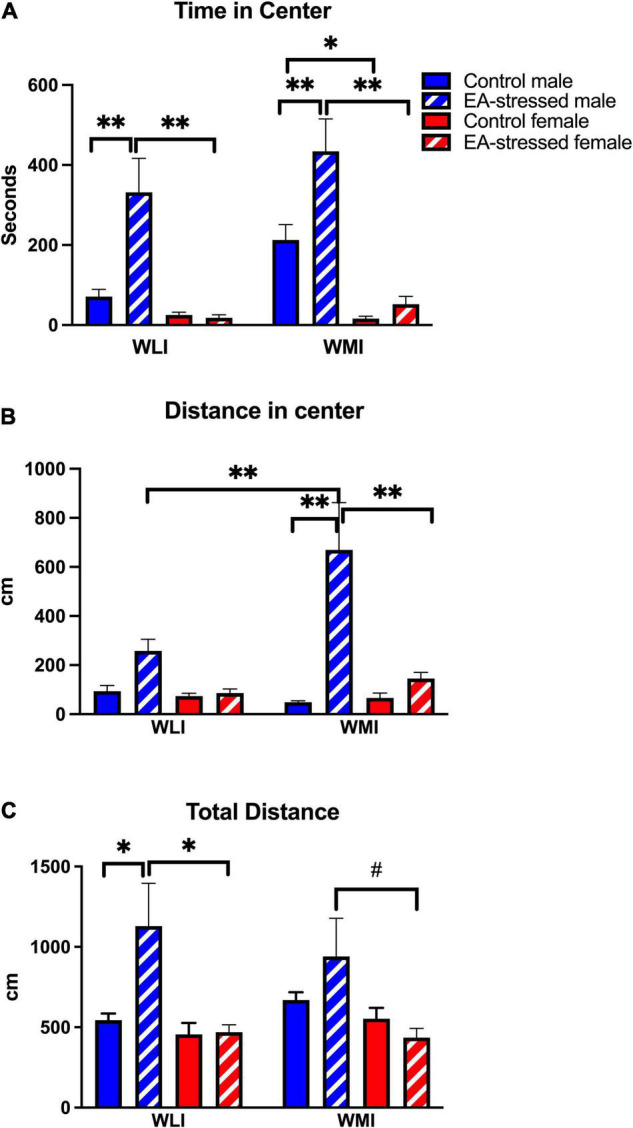
Stress during early adolescence decreases anxiety-like behavior in males of both strains compared to controls in the OFT without any effects on females. **(A)** Male WLIs and WMIs spent significantly more time in the center after stress during early adolescence (EA) compared to control males and EA-stressed females. Cohen’s d effect sizes ranged between 1.21 and 2.9. **(B)** Distance traveled by EA-stressed WMI males in the center of open field arena was significantly greater compared to control WMI males and EA-stressed WLI males and WMI females. Cohen’s d effect sizes ranged between 2.93 and 4.56. **(C)** Total distance traveled during OFT differed only by EA-stressed WLI males from control WLI males and EA-stressed WLI females. Cohen’s d effect sizes ranged between 1.04 and 1.40. Data are presented as mean ± SEM. *N* = 6–10/sex/strain/stress status. *q < 0.05; ^**^q < 0.01 by FDR *post hoc* test after three-way ANOVA. ^#^*p* < 0.05 represent independent *p*-values not corrected for multiple comparisons and shown for interpretation purposes.

Interestingly, this elevated percent freezing of adult WLI male after EA in the CFC coincided with increased time spent in the anxiety-provoking center of the OFT, but this effect was also present in EA stressed adult WMI males compared to non-stressed controls [Figure 2A; stress × sex, *F*(1, 46) = 8.06, *p* < 0.01]. Significant main effects were also seen for sex and strain [sex, *F*(1, 46) = 34.54, *p* < 0.01; stress, *F*(1, 46) = 10.23, *p* < 0.01]. There was no effect of EA stress on females of either strain.

Distance covered in the center showed a similar pattern to time in center (Figure 2B). However, it also revealed that adult WMI males after EA stress explored the center significantly more than stressed adult WLI males. Furthermore, adult stressed WMI males traveled significantly more in the center than their female counterparts after stress [strain × stress × sex, *F*(1, 49) = 4.7, *p* < 0.05; stress × sex, *F*(1, 49) = 15.0, *p* < 0.001]; [strain × stress, *F*(1, 49) = 8.6, *p* < 0.01]; [sex, *F*(1, 49) = 15.0, *p* < 0.01]; [stress, *F*(1, 49) = 24.0, *p* < 0.01]; strain, [*F*(1, 49) = 5.5, *p* < 0.05]. There was no effect of EA stress on females of either strain.

Curiously, adult EA-stressed WLI males also showed significantly increased ambulation compared to controls, which suggest that their increased distance traveled in the center are not necessarily a reflection of decreased anxiety, but more of enhanced activity (Figure 2C). Stressed WLI males also showed significantly greater activity compared to EA-stressed adult WLI females [stress × sex, *F*(1, 49) = 5.67, *p* < 0.05]; [sex, *F*(1, 49) = 11.55, *p* < 0.01]. WMIs showed no differences in total distance regardless of adolescent stress status or sex. There was no effect of EA stress on females of either strain.

**FIGURE 3:**
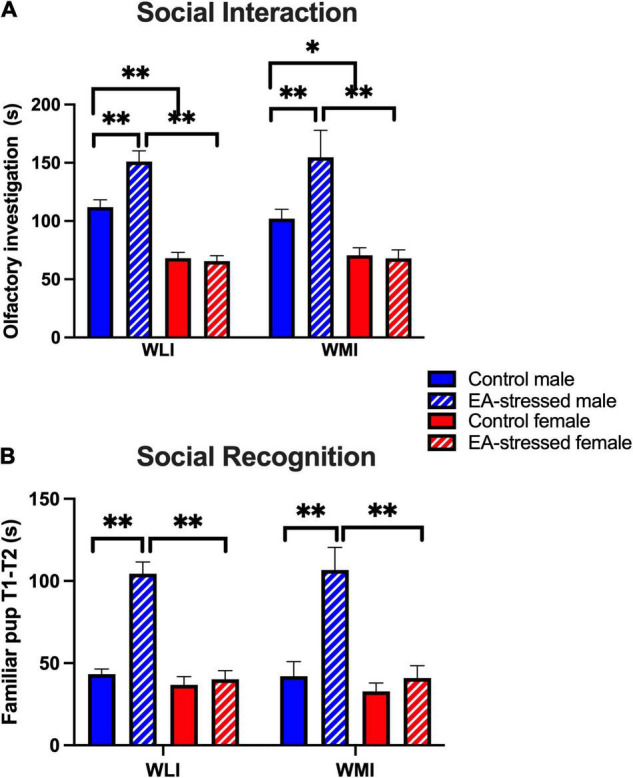
Social interaction and recognition are increased in EA-stressed males of both strains compared to controls, no change in females. **(A)** Social interaction measured by total time spent (s) with olfactory investigation of the juvenile. Cohen’s d effect sizes ranged between 3.04 and 11.7. **(B)** Social recognition or memory is characterized by the time difference in investigating the familiar pup in the first and second phases. Cohen’s d effect sizes ranged between 2.43 and 3.98. Data are presented as mean ± SEM. *N* = 4–10/sex/strain/stress status. *q < 0.05; ^**^q < 0.01 by FDR *post hoc* test after three-way ANOVA.

In the social interaction test with non-threatening juveniles, EA stressed adult males interacted significantly more than their controls regardless of strain (Figure 3A). In addition, control adult males interacted significantly more than control females and EA stressed males interacted significantly more than EA stressed females [stress × sex, *F*(1, 51) = 15.00, *p* < 0.01]; [stress, *F*(1, 51) = 12.00, *p* < 0.01]; [sex, *F*(1, 51) = 95.00, *p* < 0.01]. There was no effect of EA stress on females of either strain.

Social recognition was measured by the difference in time the animal spent with investigating the same juvenile during the original interaction when the juvenile was alone and the second interaction when there was also a novel juvenile in the cage. Increased social recognition indicated that the animals spent less time investigating the familiar animal during the second interactive test stage. Stressed adult males recognized the familiar juvenile more than control males and more than EA stressed adult females, regardless of strain [Figure 3B; stress × sex, *F*(1, 42) = 32.58, *p* < 0.01]; [stress, *F*(1, 42) = 27.54, *p* < 0.01]; [sex, *F*(1, 42) = 35.26, *p* < 0.01]. There was no effect of EA stress on females of either strain.

**FIGURE 4:**
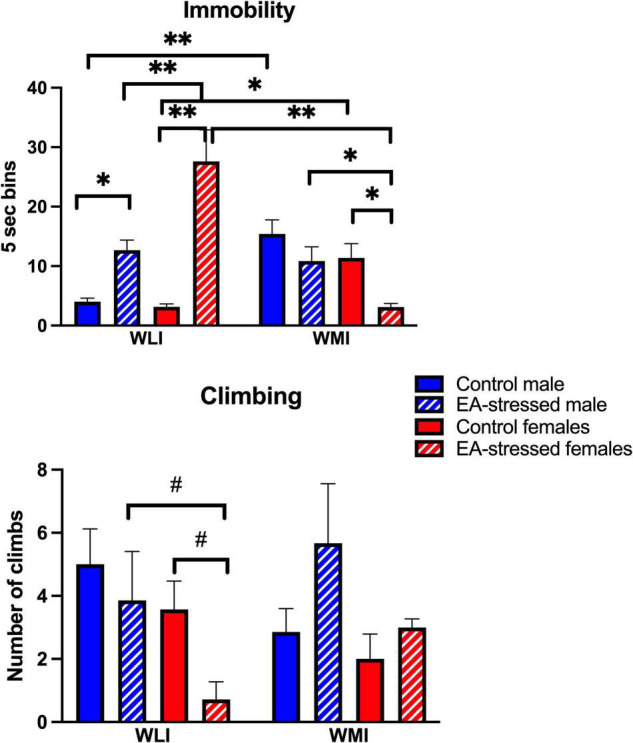
Forced swim test demonstrating immobility behavior in WMI and WLI. Early adolescent stress increases immobility behavior in adult WLIs while it reduces immobility behavior in WMI females. Immobility behavior (floating) was measured every 5s (bins) on the second day of FST. Climbing behavior was observed against the wall of the glass chamber and showed greater variations. Cohen’s d effect sizes for immobility ranged between 1.67 and 2.89. Cohen’s d effect sizes for climbing were 1.02 and 1.44. Data are presented as mean ± SEM. *N* = 4–10/sex/strain/stress status. *q < 0.05; ^**^q < 0.01 by FDR *post hoc* after three-way ANOVA. ^#^*p* < 0.05 represent independent *p*-values not corrected for multiple comparisons and shown for interpretation purposes.

The WLI and WMI inbred strains were selectively bred originally by their behavior in the FST. Thus, not surprisingly, EA stress had major, and sex-dependent effects on immobility in the FST, differently in WLIs and WMIs (Figure 4). Stressed adult WLI males and females showed significantly greater immobility than control WLIs. In contrast, adult WMI females decreased their immobility after EA stress, and no significant effects of EA stress were seen in adult WMI males [strain × sex × stress, *F* (1,51) = 8.50, *p* < 0.01]; [strain × stress, *F*(1, 51) = 48.00, *p* < 0.01]; [strain × sex, *F*(1, 51) = 15.00, *p* < 0.01]; [stress, *F*(1, 51) = 9.30, *p* < 0.01].

Climbing was not always easy to observe, and it did not reflect an inverse relationship with immobility (Figure 4). The effect of EA stress tended to decrease climbing in adult WLI females compared to controls and stressed WLI males [strain × stress, *F*(1, 47) = 7.10, *p* = 0.01]; [sex, *F*(1, 47) = 7.60, *p* < 0.01].

### Transcript Levels in the Prefrontal Cortex and the Hippocampus

**FIGURE 5:**
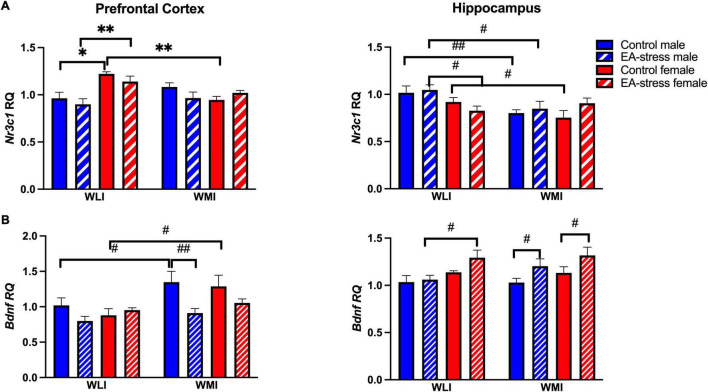
Prefrontal cortex and hippocampal expression of *Nr3c1* **(A)** and *Bdnf* **(B)** in WLI and WMI. Transcript levels were measured by quantitative RT-qPCR and shown as Relative Quantification (RQ). Data are presented as mean ± SEM. *N* = 5–7/sex/strain/stress status. *q < 0.05; ^**^q < 0.01 by FDR *post hoc* test after three-way ANOVA. ^#^*p* < 0.05, ^##^*p* < 0.01 represent independent *p*-values not corrected for multiple comparisons and shown for interpretation purposes.

Glucocorticoid receptor (*Nr3c1*) expression was significantly higher in the prefrontal cortex of control and EA-stressed adult WLI females than males, and significantly higher compared to control WMI females [Figure 5A; strain × sex × stress, *F*(1, 40) = 4.26, *p* < 0.05, sex × stress, *F*(1, 40) = 24.10, *p* < 0.01]; [strain × stress, *F*(1, 40) = 19.31, *p* < 0.01]; [strain × sex, *F*(1, 40) = 14.14, *p* < 0.01]. Analysis of hippocampal *Nr3c1* transcript levels resulted in a significant main effect of strain, although all *post hoc* tests showed only individual *p*-value significance [Figure 5A; strain, *F*(1, 40) = 8.90, *p* < 0.01]. Expression of *Nr3c1* in both control and EA-stressed adult WLI males were higher than those of WMI males, and control WLI females had greater expression than control WMI females.

*Bdnf* expression was higher in the prefrontal cortex of adult control WMIs compared to control WLIs, although EA stress eliminated this difference by reducing expression in stressed male WMIs [Figure 5B; strain, *F*(1, 39) = 9.91, *p* < 0.01]; [stress, *F*(1, 39) = 7.30, *p* = 0.01]. Interestingly, this latter difference was the opposite in the hippocampus of adult WMI males and females, who showed decreased *Bdnf* expression after EA stress [Figure 5B; stress, *F*(1, 37) = 9.96, *p* < 0.01]. Sex differences were seen between EA stressed adult WLI males and WLI females in hippocampal *Bdnf* expression [sex, *F*(1, 37) = 10.46, *p* < 0.01].

## Discussion

The goals of the study were to identify the role of genetic stress-reactivity in the long-lasting behavioral effects of stress during early adolescence. The adolescent stress was a contextual fear memory test, in which no strain and sex differences were found. The remote memory of this stress was affected in adult males, specifically in WLI males, but not in females. Similarly, adult females were not affected by EA stress in their anxiety and social behaviors, while this EA stress brought positive changes in these behaviors to adult males. However, the enhanced immobility of the less stress-reactive WLI males and females after EA stress in the FST suggest that similarly to other strains, EA stress enhances depression-like behavior or passive coping in adulthood. The opposite effect of EA stress on WMI females question whether this mild stress can generate a resilience in the stress-reactive strain, particularly in females that were not affected by EA stress in any of the other measures. The lack of effect of EA stress on *Nr3c1* expression in either brain region, but the WMI-specific changes in *Bdnf* transcript levels propose that glucocorticoid independent processes regulate these latter changes.

Adult-like CFC emerges by PD 24 (Casey et al., 2015), therefore, the lack of strain and sex differences in fear memory at EA suggests no differences in developmental trajectory between the strains. Still, despite this lack of differences in EA responses to CFC, major strain and sex differences were found in remote memory, particularly in WLI males. The increased percent freezing of adult EA-stressed WLI males is seemingly contradictory with their increased time spent in the center of the OFT. Would this mean a clear separation between fear and anxiety as increased percent freezing suggests increased fear, while increased time spent in the anxiety-provoking center of the OFT indicates decreased anxiety?

In neurobiological research, the distinction between anxiety and fear is ambiguous, and often one is used to define the other (Perusini and Fanselow, 2015). Fear is thought to be specific to responses directed to a present threat associated with specific cues or contexts, while anxiety is in preparation for threats that are future-oriented (Davis et al., 2010). The distinct definition of fear and anxiety are supported by opposing changes found after environmental challenges in these two facets of danger-induced behaviors (Heinz et al., 2021). However, human and animal studies support the opposing hypothesis showing correlations between these behaviors (Indovina et al., 2011; Ahn et al., 2013). Since anxiety measurements are based on the rodents’ natural tendency to avoid open areas, it is thought to measure intrinsic anxiety-related characteristics of the animal. Since male WMIs already show less anxiety than WLIs (Mehta et al., 2013), the exaggerated effect of EA stress on decreasing anxiety of the adult WMI males could be due this innate difference. Indeed, the genetically high anxiety HR rats also show increased contextual fear memory compared to the low anxiety LR animals (Lehner et al., 2010), just like the EA-stressed WLI males.

The finding of decreased anxiety-like behaviors in males of both strains after EA stress is similar to some, but dissimilar to other findings in the literature. Acute lipopolysaccharide injection at PND 26 shown to be anxiolytic in male, but not in female, Wistar rats as measured by time spent in the open arm of the elevated plus maze (EPM) (Ariza Traslaviña et al., 2014). No other acute stressor applied at the same developmental period showed this response in the study. Sex specific effect is also reported for Wistar rats, when acute stress during adolescence increases anxiety behavior in male Sprague-Dawley rats, while females are not affected (Lovelock and Deak, 2019). Most studies report increased anxiety-like behaviors in adult male rodents after a single or repeated mild stress during the same developmental period (Tsoory et al., 2007; Mancini et al., 2021; Meyer et al., 2021). In contrast, a 3-day stress in the same EA period increases anxiety-like measures in the OFT and the EPM in both adult male and female rats (Jacobson-Pick and Richter-Levin, 2010).

The current findings of decreased anxiety-like behaviors in adult males after EA stress is novel and at variance with other studies. The cause of these differing results is likely related to the strains of the animals employed. The parental strain of the WLI and WMI, the WKY strain, show increased anxiety and depression-like behavior as measured by immobility in the FST. However, immobility in the FST is also considered to be more of a measure of passive coping with stress (de Kloet and Molendijk, 2016). The selective breeding was based on the immobility behavior, and anxiety behavior in the OFT did not segregate consistently between the strains. In the present study, males of both strains showed decreased adult anxiety-like behavior after the relatively mild acute stress during early adolescence, suggesting a resilience generated by the EA stress. In contrast, only adult WMI females had a positive effect of adolescent stress on immobility in the FST. Interestingly, in a recent study we found that stress during adolescence in another strain of rats with genetic predisposition to increased passive coping led to increased anxiety in males, but decreased immobility in the FST in females (Ji et al., 2021). Would females genetically predisposed to increased passive coping be more resilient to the effects of adolescent stress than males of the same strain? Would the resilience generated in females after the double hit of adolescent stress and the stress of FST in adulthood be like the resilience generated by social defeat in adolescence to the single stress in adulthood (Mancini et al., 2021)? Future studies modifying the nature of the stressor in adolescence and the strain of animals used could in part answer this question.

The increased social interaction and recognition observed in adult male rats after EA stress may be related to the decreased anxiety-like behavior observed. This hypothesis could be confirmed if other genetically anxious or stress-prone strains show similar phenomena. The Roman Low Avoidance (RLA) strain has consistently shown high anxiety compared to the Roman High Avoidance (RHA) animals (Giorgi et al., 2019). However, after neonatal handling, social interaction increases in males of both strains in parallel with their decreased anxiety (Sampedro-Viana et al., 2021). The authors of that study argue that the effects of neonatal handling on social interaction seem to be independent from the reduction of anxiety. In a similar vein, we could argue that these phenotypes are also independent in our study as anxiety-like behavior as measured by distance traveled in the center of the OFT is less improved in EA-stressed WLI males compared to WMI males. Still social interaction is improved by EA stress to a similar degree in males of both strains.

Females of either strain were resistant to the behavioral changes caused by EA stress in agreement with many studies, using similar or dissimilar stressors and prepubertal or adolescent developmental time frame (Klinger et al., 2019). Other studies have found no effect in females (different strains, stressors and ages of exposure to stress) in the EPM and/or in the OFT (Barna et al., 2003; Slotten et al., 2006; Prusator and Greenwood-Van Meerveld, 2015; Guadagno et al., 2018). Although examined at earlier time points (i.e., PD3, PD9, or PD11) and different stressors, others have also found no impact of early life stress on female rats with respect to the EPM, NOR, and object recognition location test (Loi et al., 2017). A recent study conducted in female MAM rats also found no differences in anxiety-like behavior in the EPM (Perez et al., 2019).

The passive coping measure of immobility responded to EA stress in both adult males and females. EA stress increased immobility in the adult WLI males, similarly to our findings after chronic stress in adulthood (Mehta-Raghavan et al., 2016). However, it did have the opposite effect on adult females; increased immobility in WLIs, but decreased immobility in WMI females after EA stress. While one can argue that in the case of males, control WMI males show a ceiling effect in immobility, this is clearly not the case for females, as immobility of EA-stressed WLI females is higher than that of control WMI females. The exaggerated immobility of stressed WLI females is inverse of their decreased climbing behavior, confirming this finding. As the WLI and WMI inbred strains were originally selected for immobility behavior in the FST, these changes induced by EA stress suggest that in the absence of genetic predisposition, even a minor stress during early adolescence could have adverse long-lasting effects on the individual. However, the same minor stress could generate resilience in the genetically predisposed individual. A recent very illuminating review of resilience discuss novel interpretation of resilience, and pathways that can lead to it (Malhi et al., 2019).

Brain-derived neurotrophic factor is a growth factor acting through TrkB tyrosine kinase receptors to promote neuronal survival and differentiation. The evidence for a critical role of BDNF in resilience during development is largely based on animal models of chronic stress (Taliaz et al., 2011), which significantly implicate hippocampal BDNF mediation. BDNF genes are also involved in the development of neural circuits that control coping mechanisms (Cattaneo et al., 2015). BDNF is also involved in social behaviors as demonstrated by animal models of social behaviors (Branchi et al., 2013). It is also critical for experience-dependent synaptic plasticity and memory, including fear learning (Chao, 2003; Lee et al., 2003; Andero and Ressler, 2012; Casey et al., 2015). Loss of *Bdnf* expression in adult genetic knockout mice leads to impaired fear learning and increased anxiety-related behaviors (Chen et al., 2006). Furthermore, *Bdnf* expression in the hippocampus is linearly correlated with time spent in the center of the OFT (Bahi, 2017). Thus, we expected that at least some of the behavioral phenotypes show correlations with *Bdnf* expression. This was not the case. It is proposed that decreased *Bdnf* does not cause depressive behaviors but does hamper the effect of antidepressant drugs (Martinowich et al., 2007), therefore, the lack of correlation between *Bdnf* expression and the behavioral phenotypes may only reflect that. There was also no correlation found between the expression of the glucocorticoid receptor and that of *Bdnf* in the same brain regions. However, both *Nr3c1* and *Bdnf* expression were measured after the series of behavioral tests, which could clearly obscure any effects of EA stress on the expression of these genes.

This study is novel, as for the first time it demonstrates the complex effect of early adolescent stress on two nearly isogenic inbred strains exhibiting high, and low stress-reactivity. The sex-specific findings are also very illuminating, particularly the major sex differences in vulnerability to EA stress. One caveat of our study is the nature and the timing of the stress employed. Stronger and different, for example metabolic, stressors may lead to different outcomes. Additionally, despite our efforts to eliminate carryover effects of the different behavioral measurements, a cumulative stress affecting the adult animals could not be excluded. Future studies can investigate these questions and characterize whether the observed sex differences are related to differences in the early adolescent neurodevelopmental stages between males and females. Additionally, further exploration is needed to determine if the resilience caused by the EA stress can be generated at different time frames or by different stressors in both sexes.

## Data Availability Statement

The original contributions presented in the study are included in the article/supplementary material, further inquiries can be directed to the corresponding author/s.

## Ethics Statement

The animal study was reviewed and approved by Institutional Animal Care and Use Committee of Northwestern University.

## Author Contributions

SK, MM, and SG: experimental work, analyzing, interpreting data, and editing the manuscript. ER: conceptualization, writing editing of manuscript, analyzing, and interpreting data. All authors contributed to the article and approved the submitted version.

## Conflict of Interest

The authors declare that the research was conducted in the absence of any commercial or financial relationships that could be construed as a potential conflict of interest.

## Publisher’s Note

All claims expressed in this article are solely those of the authors and do not necessarily represent those of their affiliated organizations, or those of the publisher, the editors and the reviewers. Any product that may be evaluated in this article, or claim that may be made by its manufacturer, is not guaranteed or endorsed by the publisher.
